# Exploring the Immunological Shield Hypothesis: A Population-Based Exploration of Phenotypic Divergence Between Lipedema and Celiac Disease Autoimmunity

**DOI:** 10.7759/cureus.104222

**Published:** 2026-02-25

**Authors:** Alexandre C Amato, Juliana L Amato, Daniel Benitti

**Affiliations:** 1 Department of Vascular Surgery, Amato - Instituto de Medicina Avançada, São Paulo, BRA; 2 Department of Gynecology, Amato - Instituto de Medicina Avançada, São Paulo, BRA; 3 Department of Vascular and Endovascular Surgery, Medical Valens Center, São Paulo, BRA

**Keywords:** adiponectin, autoimmunity, celiac disease, gluteofemoral adipose tissue, homa-ir, immunometabolism, lipedema, nhanes

## Abstract

Background

Lipedema is characterized by disproportionate gluteofemoral adiposity with anti-inflammatory properties. We hypothesized that this phenotype may confer immunological protection against T-helper 1 (Th1)-mediated autoimmunity ("Immunological Shield Hypothesis").

Objective

The objective of this study is to explore whether women with a dual-energy X-ray absorptiometry (DXA)-defined lipedema-like phenotype, characterized by disproportionate gluteofemoral fat accumulation, exhibit distinct immunometabolic profiles and lower prevalence of celiac disease (CD) autoimmunity in a nationally representative sample.

Methods

The cross-sectional analysis included 3,833 women from the National Health and Nutrition Examination Survey (NHANES) 2011-2014. Celiac disease (n=11, 0.56% weighted prevalence) was defined by strict serology (tissue transglutaminase {tTG}-IgA+/endomysial antibody {EMA}-IgA+); lipedema phenotype was defined as leg-to-trunk fat ratio of >90th percentile via DXA.

Results

Women with celiac disease exhibited 7.4% lower gynoid fat (39.5% versus 42.6%, p=0.0007), persisting in overweight/obese strata. Conversely, the lipedema phenotype demonstrated superior metabolic health: 44.2% lower homeostatic model assessment of insulin resistance (HOMA-IR) (p<0.001) and 7.6% lower neutrophil-to-lymphocyte ratio (NLR) (p=0.012).

Conclusions

This exploratory population-based analysis identifies phenotypic divergence in fat distribution between the DXA-defined lipedema phenotype and celiac disease autoimmunity, yielding observations consistent with, but not confirmatory of, the "Immunological Shield Hypothesis." While limited by the small number of celiac cases (n=11), a sample size insufficient to detect prevalence differences for a ~7%-9% phenotype, for which approximately 225-600 celiac cases would be required, the observed differences in gynoid adiposity (7.4% reduction, p=0.0007) and the favorable metabolic profile of the lipedema phenotype (44.2% lower HOMA-IR and 7.6% lower NLR) suggest biological plausibility warranting validation in larger, targeted cohorts. These findings motivate targeted studies to evaluate whether dietary exposures, including gluten-related immune activation, interact with gluteofemoral adipose biology in lipedema.

## Introduction

Lipedema is a chronic, progressive disorder of adipose tissue characterized by the disproportionate and symmetrical accumulation of subcutaneous fat in the lower extremities, often sparing the feet and trunk [1,2]. Although frequently misdiagnosed as general obesity or lymphedema, lipedema constitutes a distinct clinical entity with a unique pathophysiology involving microvascular dysfunction, interstitial fluid accumulation, and chronic low-grade inflammation. Unlike common obesity, the adipose tissue in lipedema is resistant to diet and exercise-induced weight loss, suggesting a distinct metabolic and hormonal regulation profile [3,4].

While the inflammatory component of lipedema is well-recognized, its intersection with systemic autoimmune conditions remains poorly understood [5-14]. Celiac disease (CD) is an immune-mediated enteropathy triggered by the ingestion of gluten in genetically susceptible individuals, characterized by a T-helper 1 (Th1)-driven inflammatory response. Autoimmune diseases typically exhibit a tendency to cluster; however, clinical observations suggest that the distinct immunometabolic milieu of lipedema, potentially characterized by a different cytokine profile or adipose-derived modulation, might differ significantly from the classical autoimmune phenotype associated with CD [10,13,15-17]. To date, no epidemiological studies have investigated the potential relationship between these two conditions.

Investigating this association on a population level presents a significant methodological challenge: lipedema remains underdiagnosed and lacks a specific International Classification of Diseases (ICD) code in many historical datasets. However, recent studies have validated the utility of dual-energy X-ray absorptiometry (DXA) in identifying "lipedema-like" phenotypes based on regional fat distribution indices, such as the leg-to-trunk fat ratio [18,19]. These imaging biomarkers distinguish the disproportionate lower-body adiposity characteristic of lipedema from the generalized or android fat distribution typical of common obesity.

In this study, we utilized data from the National Health and Nutrition Examination Survey (NHANES) to explore whether women exhibiting a DXA-defined lipedema-like phenotype have a lower prevalence of celiac disease autoimmunity [20]. It is important to note that the "lipedema phenotype" used throughout this study refers to a DXA-derived proxy definition based on the >90th percentile of the leg-to-trunk fat mass ratio and does not constitute a clinical diagnosis of lipedema. This proxy may capture normal constitutional variation in fat distribution (e.g., gynoid obesity) rather than true lipedema pathology. By leveraging whole-body DXA scans and serological testing from a nationally representative sample, our primary objective was to compare body composition and celiac disease prevalence across phenotypic groups, while the secondary objective was to characterize immunometabolic profiles associated with the DXA-defined lipedema proxy. Analyses within body mass index (BMI) and ethnicity subgroups were conducted to assess robustness [13].

Given the challenges of studying lipedema (the lack of specific diagnostic codes and clinical underdiagnosis) and the low population prevalence of celiac disease (~0.5%), this study serves as an exploratory, hypothesis-generating investigation. We acknowledge a priori that the expected small number of celiac cases in a nationally representative sample limits definitive conclusions but may reveal biologically meaningful patterns warranting validation in larger, targeted cohorts.

## Materials and methods

Study design and population

This cross-sectional study utilized data from the NHANES 2011-2014 cycles. NHANES is a nationally representative survey of the noninstitutionalized US population conducted by the National Center for Health Statistics (NCHS). The survey employs a complex, multistage probability sampling design and collects demographic, dietary, examination, and laboratory data. All protocols were approved by the NCHS Research Ethics Review Board (approval number: 2011-17), and the participants provided written informed consent.

**Figure 1:**
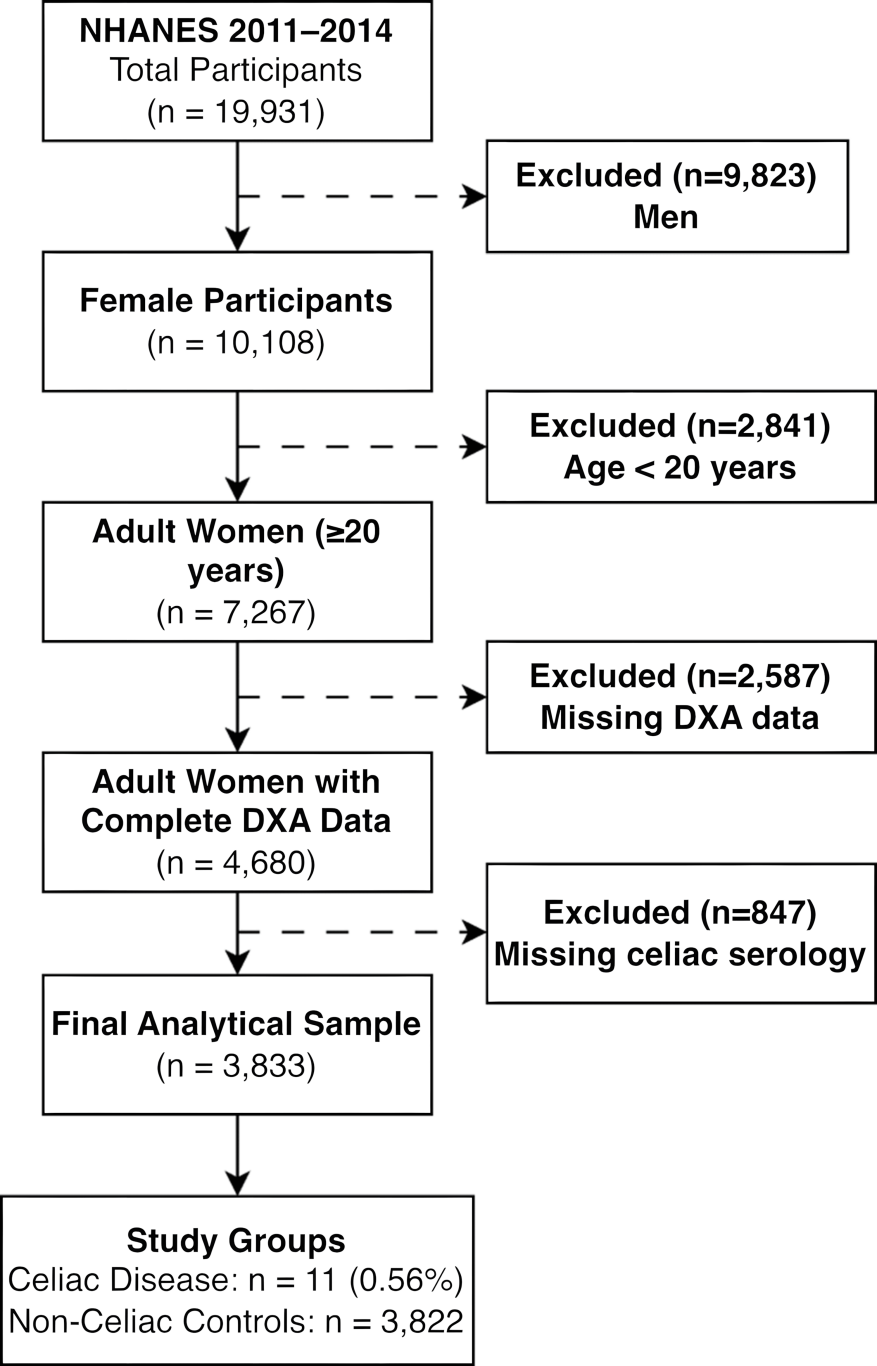
Participant Flowchart Illustrating the Selection Criteria From the NHANES 2011-2014 Database. NHANES, National Health and Nutrition Examination Survey; DXA, dual-energy X-ray absorptiometry

We included adult women aged 20 years and older with complete data for DXA body composition assessment and celiac disease serology. Men were excluded as lipedema predominantly affects women. The participants missing key exposure or outcome variables were excluded from the analysis (see Appendices and Figure 1).

Celiac disease definition

Celiac disease was defined serologically using a strict dual-positive criterion requiring both tissue transglutaminase (tTG)-IgA antibody positivity and endomysial antibody (EMA)-IgA positivity. This definition represents the gold standard serological approach for population-based studies and minimizes false-positive classifications.

Body composition assessment

Body composition was assessed using whole-body DXA scans performed with Hologic Discovery model A densitometers (Hologic, Inc., Bedford, MA). Regional fat mass measurements were obtained for the legs (left and right combined), trunk, android region, and gynoid region. The primary exposure variable was gynoid region percent fat, representing the percentage of fat in the hip and thigh region. Secondary measures included the leg-to-trunk fat ratio, calculated as total leg fat mass divided by trunk fat mass, and the android-to-gynoid ratio, representing the ratio of android-to-gynoid percent fat. The lipedema phenotype was operationally defined as a leg-to-trunk fat ratio exceeding the 90th percentile of the female population distribution, representing women with disproportionately high lower-body fat accumulation.

Height and weight were measured using standardized protocols. BMI was calculated as weight in kilograms divided by height in meters squared. Waist circumference was measured at the iliac crest, and the waist-to-height ratio was calculated as waist circumference divided by height.

Key NHANES variables included the following: DXA regional fat measures from DXX_G and DXX_H data files (e.g., DXXLAFAT and DXXLLFAT for limb fat, DXDTRFAT for trunk fat, and DXDGPFAT for gynoid percent fat), celiac serology from TGEMA_G/H (LBDTTG for tTG-IgA and LBDEMA for EMA), complete blood count from CBC_G/H (LBXWBCSI for white blood cell {WBC}, LBXNEPCT for neutrophil percent, and LBXLYPCT for lymphocyte percent), fasting insulin from INS_G/H (LBXIN), and fasting glucose from GLU_G/H (LBXGLU). The neutrophil-to-lymphocyte ratio (NLR) was calculated as LBXNEPCT/LBXLYPCT. The homeostatic model assessment of insulin resistance (HOMA-IR) was calculated as (LBXIN×LBXGLU)/405.

Immunometabolic markers

To further characterize the lipedema phenotype, we conducted an exploratory analysis of immunometabolic markers. The neutrophil-to-lymphocyte ratio (NLR), a validated marker of systemic inflammation, was calculated from complete blood count data as neutrophil percentage divided by lymphocyte percentage. Insulin resistance was assessed using the homeostatic model assessment (HOMA-IR), calculated as fasting glucose multiplied by fasting insulin divided by 405, where glucose is expressed in mg/dL and insulin in µU/mL. Secondary exploratory analyses validating the immunometabolic profile against other conditions are presented in the Appendices.

Statistical analysis

All primary analyses incorporated NHANES complex survey design weights to ensure nationally representative estimates. Four-year mobile examination center (MEC) examination weights were constructed by dividing the two-year weights (WTMEC2YR) by 2, per NHANES analytic guidelines for combined survey cycles. Variance estimation employed Taylor series linearization, accounting for masked variance primary sampling units (SDMVPSU) and strata (SDMVSTRA) as provided in the NHANES demographic files. Unweighted statistics (sample sizes, exact counts, and Fisher's exact tests) are reported for sample characterization where explicitly noted. Continuous variables were compared using survey-weighted t-tests; categorical associations were tested using Fisher's exact test due to small cell counts in the celiac group (n=11). Analyses were conducted as complete-case analyses within each analytic domain. The participants missing valid DXA data (due to pregnancy, hardware limitations, or scan artifacts flagged by NHANES quality codes) or celiac serology were excluded. The analytic sample represents the participants with complete data for both DXA body composition and dual celiac serology (see participant flow chart, Appendices and Figure 1). The Mann-Whitney U test was used for skewed distributions. Statistical significance was set at p<0.05. All software used is freely available under open-source licenses: Python 3.12 (Python Software Foundation {PSF} License), pandas (Berkeley Software Distribution 3 {BSD-3}), SciPy (BSD-3), and statsmodels (BSD-3). Complete analytic code (Python) is available from the corresponding author upon reasonable request.

To address the potential concern that lower gynoid fat in celiac patients might reflect overall leanness or malnutrition, we conducted a sensitivity analysis restricted to overweight and obese women (BMI>25 kg/m²). Additionally, we stratified by BMI category (normal weight, 18.5-24.9; overweight, 25-29.9; and obese, ≥30 kg/m²) to assess whether the association persisted across the weight spectrum.

## Results

A total of 3,833 adult women with complete DXA and celiac serology data were included. Among these, 11 women met the strict serological criteria for celiac disease, representing a weighted prevalence of 0.56%. Women with celiac disease were similar in BMI (27.7±8.0 versus 29.2±7.9 kg/m², p=0.390) but were significantly more likely to be non-Hispanic White (81.8% versus 36.2%, p=0.003) (Table 1).

**Table 1:** Characteristics of the Study Population by Celiac Disease Status (Survey-Weighted). Data are presented as survey-weighted mean ± SD or n (%) (NHANES 2011-2014). Continuous variables compared using Welch's t-test (survey-weighted, unequal variances assumed). Celiac disease defined as tTG-IgA-positive and EMA-IgA-positive. Lipedema phenotype defined as a leg-to-trunk fat ratio of >90th percentile. †Fisher's exact test (unweighted due to small cell counts). BMI, body mass index; SD, standard deviation; NHANES, National Health and Nutrition Examination Survey; tTG, tissue transglutaminase; EMA, endomysial antibody

Characteristic	Non-celiac (n=3,822)	Celiac Disease (n=11)	Test Statistic	P-value
BMI, kg/m²	29.2±7.9	27.7±8.0	t=-0.88	0.390
Leg fat mass, kg	11.7±4.4	9.3±1.9	t=-5.45	<0.001
Leg-to-trunk fat ratio	0.89±0.26	0.77±0.26	t=-2.19	0.040
Gynoid region percent fat	42.6±5.2	39.5±3.6	t=-3.96	0.0007
Android region percent fat	38.7±8.4	37.0±7.0	t=-1.11	0.282
Android/gynoid ratio	0.917±0.153	0.927±0.142	t=0.23	0.820
Lipedema phenotype, n (%)	282 (7.4)	1 (9.1)	-	0.570†
Non-Hispanic White, n (%)	1,383 (36.2)	9 (81.8)	-	0.003†

**Figure 2:**
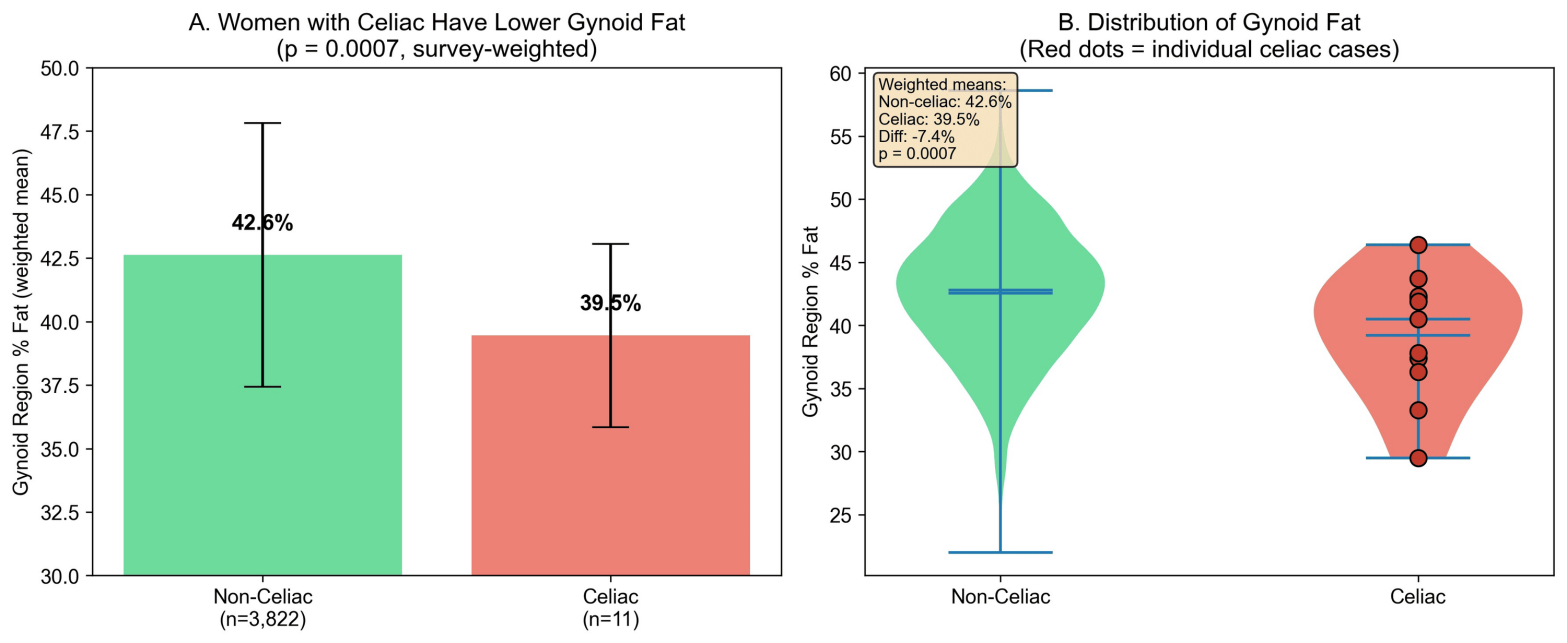
Women With Celiac Disease Have Significantly Lower Gynoid Fat. (A) Bar chart comparing mean gynoid region percent fat between women without celiac disease (n=3,822) and women with serologically confirmed celiac disease (n=11). Error bars represent standard deviation. (B) Violin plots with overlaid box plots showing the distribution of gynoid percent fat in both groups. Red diamond indicates group means. Women with celiac disease had 7.4% lower gynoid fat (p=0.0007). NHANES 2011-2014, adult women aged 20 years and older. NHANES: National Health and Nutrition Examination Survey

**Figure 3:**
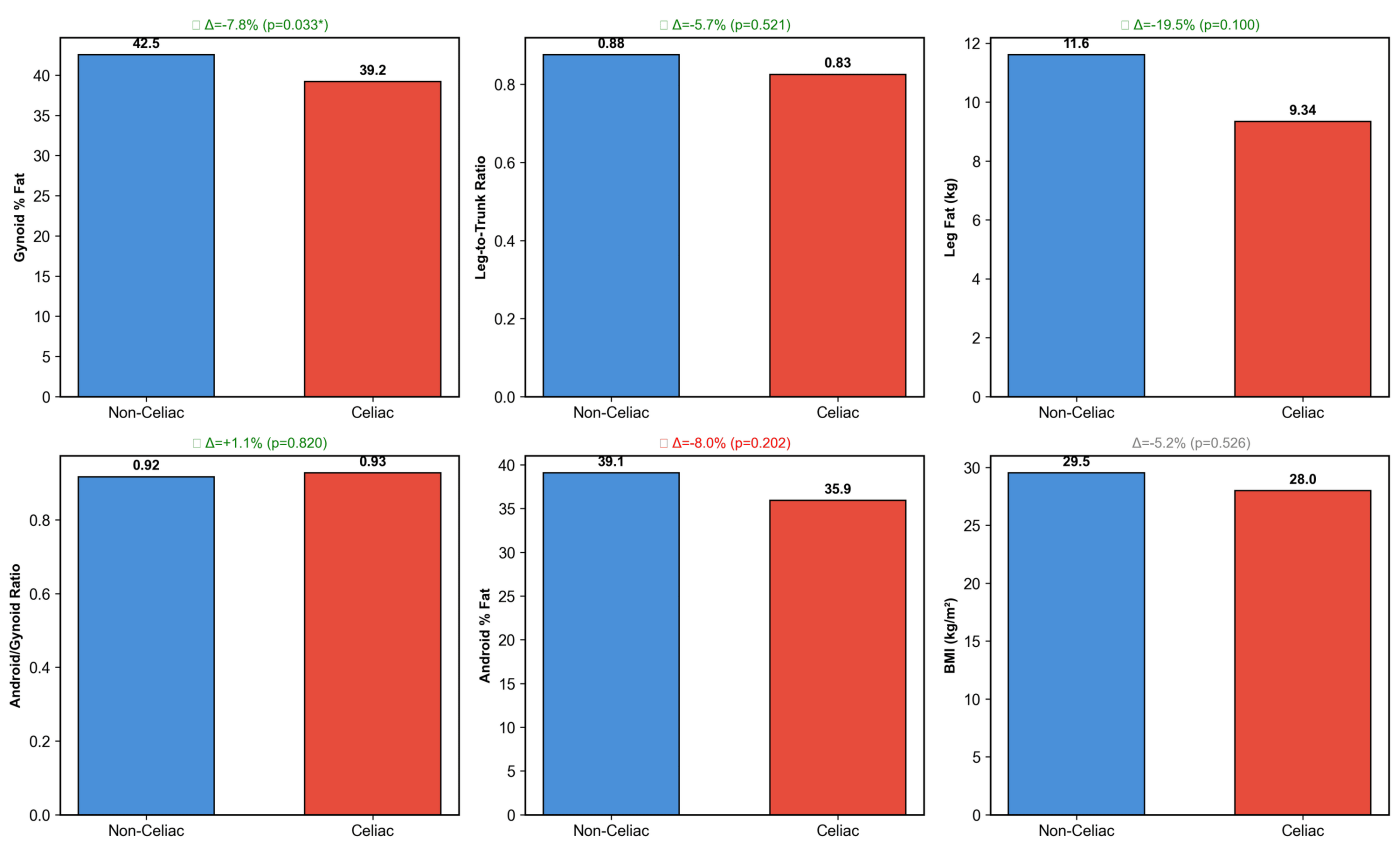
Comparison of Multiple Lipedema Phenotype Proxies by Celiac Disease Status. Six body composition measures were compared between women with and without celiac disease. Green marks indicate proxies where the direction of difference supports the hypothesis that celiac disease is associated with less gynoid fat. Only the gynoid region percent fat reached statistical significance (p=0.0007). NHANES 2011-2014, adult women aged 20 years and older. *Statistically significant difference (p<0.05). NHANES, National Health and Nutrition Examination Survey; BMI, body mass index

Women with celiac disease had significantly lower gynoid region percent fat (39.5%±3.6% versus 42.6%±5.2%, p=0.0007), representing a 7.4% relative reduction (Table 2 and Figure 2). Women with celiac disease also had a 14.1% lower leg-to-trunk fat ratio (0.77±0.26 versus 0.89±0.26, p=0.040) and 20.3% less leg fat mass (9.3±1.9 kg versus 11.7±4.4 kg, p<0.0001). The android-to-gynoid ratio was similar between groups (p=0.820), suggesting a preferential reduction in gynoid fat (Figure 3). Android region percent fat was also similar (37.0%±7.0% versus 38.7%±8.4%, p=0.282).

**Table 2:** Comparison of Lipedema Phenotype Proxies by Celiac Disease Status (Survey-Weighted). Data are presented as survey-weighted mean±SD. Bold indicates statistically significant differences (p<0.05). All proxies derived from DXA except BMI (anthropometry). P-values from Welch's t-test (survey-weighted, unequal variances assumed). Bold text indicates statistically significant differences (p<0.05). BMI, body mass index; DXA, dual-energy X-ray absorptiometry; SD, standard deviation

Proxy	Non-celiac	Celiac Disease	Difference	Test Statistic	P-value
Gynoid region percent fat	42.6±5.2	39.5±3.6	-7.4%	t=-3.96	0.0007
Leg-to-trunk fat ratio	0.89±0.26	0.77±0.26	-14.1%	t=-2.19	0.040
Leg fat mass, kg	11.7±4.4	9.3±1.9	-20.3%	t=-5.45	<0.001
Android/gynoid ratio	0.92±0.15	0.93±0.14	+1.1%	t=0.23	0.820
Android region percent fat	38.7±8.4	37.0±7.0	-4.4%	t=-1.11	0.282
BMI, kg/m²	29.2±7.9	27.7±8.0	-5.3%	t=-0.88	0.390

**Figure 4:**
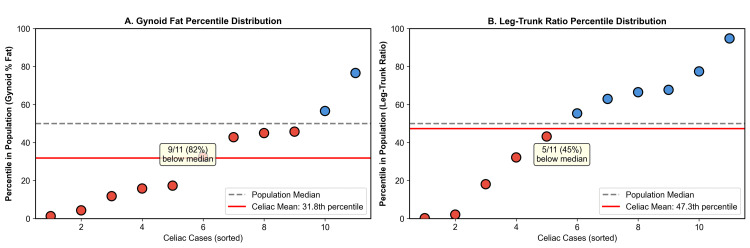
Distribution of Celiac Cases Within the Population. (A) Gynoid percent fat percentile distribution of the 11 celiac cases. (B) Leg-to-trunk fat ratio percentile distribution. Red points indicate cases below the population median; blue points indicate cases above the median. The dashed gray line represents the 50th percentile, and the solid red line represents the mean percentile of celiac cases. The majority of celiac cases cluster below the population median for gynoid fat measures.

**Figure 5:**
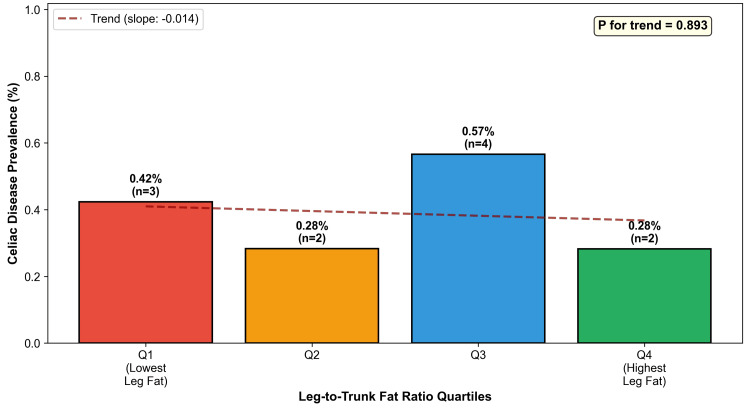
Celiac Disease Prevalence by Fat Distribution Quartiles. Bar chart showing celiac disease prevalence across quartiles of leg-to-trunk fat ratio. No significant dose-response relationship was observed (P for trend=0.893), likely due to the small number of celiac cases (n=11).

Among 11 women with celiac disease, only one (9.1%) met the criteria for lipedema phenotype compared to 282 (7.4%) without celiac disease (p=0.570). No celiac cases fell within the most extreme gynoid fat category (android-to-gynoid ratio of <10th percentile). Seven of 11 (64%) celiac cases had gynoid percent fat below the population median (Figure 4). Celiac prevalence across leg-to-trunk fat ratio quartiles showed no clear dose-response pattern (p for trend=0.893) (Table 3 and Figure 5).

**Table 3:** Celiac Disease Prevalence by Leg-to-Trunk Fat Ratio Quartiles. P for trend=0.893; Spearman rho=-0.002 (Spearman rank correlation test).

Quartile	Ratio Range	Mean Ratio	N	Celiac Cases	Prevalence
Q1 (lowest)	0.22-0.69	0.584	708	3	0.42%
Q2	0.69-0.84	0.765	707	2	0.28%
Q3	0.84-1.02	0.926	707	4	0.57%
Q4 (highest)	1.02-2.55	1.228	708	2	0.28%

Among celiac cases, none were underweight, and seven (63.6%) were overweight or obese. When restricted to overweight/obese women (BMI>25), those with celiac disease had significantly lower gynoid fat (40.6%±3.6% versus 44.5%±4.6%, p=0.005), an 8.7% reduction. The difference was most pronounced in obese women (11.3% reduction, p=0.039), ruling out malnutrition as a confounding factor.

**Figure 6:**
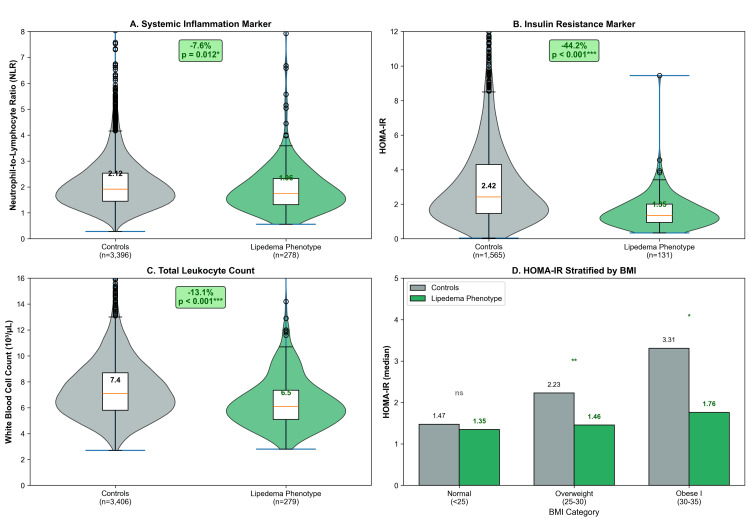
Exploratory Immunometabolic Profile: Lipedema Phenotype Versus Controls. (A) Neutrophil-to-lymphocyte ratio (NLR), a marker of systemic inflammation, was significantly lower in women with the lipedema phenotype (p=0.012). (B) HOMA-IR, a marker of insulin resistance, was markedly lower in the lipedema phenotype group (p<0.001). (C) Total white blood cell count was significantly reduced in women with the lipedema phenotype (p<0.001). (D) HOMA-IR stratified by BMI category, demonstrating that the favorable metabolic profile persists across weight categories, with the largest differences observed in overweight and obese women. Green bars indicate the lipedema phenotype group; gray bars indicate controls. NHANES 2011-2014, adult women aged 20 years and older. ​*P<0.05. **P<0.01. ***P<0.001. HOMA-IR, homeostatic model assessment of insulin resistance; BMI, body mass index; NHANES, National Health and Nutrition Examination Survey; ns, not significant (p≥0.05)

Women with the lipedema phenotype (n=283) exhibited significantly lower systemic inflammation: NLR was 7.6% lower (1.96±1.04 versus 2.12±1.03, p=0.012), and WBC was 13.1% lower (6.47±2.05 versus 7.44±2.31×10³/µL, p<0.001). Median HOMA-IR was 44.2% lower (1.35 versus 2.42, p<0.001) (Table 4 and Figure 6). This metabolic advantage increased with BMI: 34.7% lower HOMA-IR in overweight women (p=0.002) and 46.7% lower in class I obesity (p=0.019).

**Table 4:** Immunometabolic Profile by Lipedema Phenotype Status. Data are presented as mean±SD or median (IQR). NLR and WBC compared using Welch's t-test (survey-weighted); HOMA-IR compared using the Mann-Whitney U test due to skewed distribution. Lipedema phenotype defined as a leg-to-trunk fat ratio of >90th percentile. NLR, neutrophil-to-lymphocyte ratio; HOMA-IR, homeostatic model assessment of insulin resistance; WBC, white blood cell; BMI, body mass index; SD, standard deviation

Marker	Controls (n=3,550)	Lipedema Phenotype (n=283)	Difference	Test Statistic	P-value
NLR	2.12±1.03	1.96±1.04	-7.6%	t=-3.31	0.012
HOMA-IR	2.42 (1.47-4.30)	1.35 (0.95-2.01)	-44.2%	U=53,696	<0.001
WBC, 10³/µL	7.44±2.31	6.47±2.05	-13.1%	t=-8.00	<0.001
HOMA-IR by BMI					
Normal (<25 kg/m²)	1.47 (1.03-2.12)	1.35 (0.93-1.86)	-8.7%	U=22,055	0.116
Overweight (25-30)	2.23 (1.51-3.54)	1.46 (1.07-2.38)	-34.7%	U=2,145	0.002
Obese I (30-35)	3.31 (2.15-5.22)	1.76 (1.34-2.95)	-46.7%	U=443	0.019

## Discussion

This study presents the first epidemiological analysis contrasting the body composition of patients with celiac disease against the lipedema-like phenotype in a nationally representative sample. Our findings reveal a phenotypic divergence: women with celiac disease exhibited reduced gynoid adiposity (p=0.0007), whereas the DXA-defined lipedema phenotype was associated with lower insulin resistance (HOMA-IR) and systemic inflammation (NLR). These observations suggest that these conditions may occupy different positions along the immunometabolic spectrum, though the cross-sectional design precludes causal inference.

The difference in lipedema phenotype prevalence between groups did not reach statistical significance (p=0.570), and no dose-response trend was observed across leg fat quartiles (p=0.893). These findings should be interpreted in the context of the severely limited statistical power afforded by only 11 celiac cases, which is insufficient to detect prevalence differences for a phenotype present in ~7%-9% of the population. Notably, the data do show a consistent and significant pattern at the tissue level: gynoid fat percentage was markedly lower in celiac women (p=0.0007), an effect that intensified rather than attenuated in overweight and obese strata, arguing against confounding by leanness. While these tissue-level differences are consistent with the proposed hypothesis, we acknowledge that a direct protective effect of the lipedema phenotype on celiac autoimmunity was not demonstrated in this sample and remains to be tested in adequately powered cohorts. Post hoc power calculations indicate that detecting a 50% reduction in lipedema phenotype prevalence among celiac patients (from ~7.4% to ~3.7%) with 80% power would require approximately 225-600 celiac cases, underscoring that the present null finding is a function of sample size, not effect size.

Biological plausibility

This immunometabolic distinction may offer a framework for understanding the reported clinical utility of ketogenic diets for lipedema [21]; one untested hypothesis is that the therapeutic benefit may stem not only from ketogenesis but also from gluten exclusion, potentially mitigating inflammatory triggers. This pathway was not directly measured in the present study.

Celiac disease is driven by a Th1-dominant inflammatory response. Conversely, gluteofemoral adipose tissue acts as a "metabolic sink," secreting higher levels of adiponectin, an anti-inflammatory adipokine known to suppress Th1 differentiation [22-26]. The expansion of this subcutaneous depot may create a systemic anti-inflammatory milieu that raises the threshold for autoimmune activation. While these observations are directionally consistent with the hypothesis that gluteofemoral adiposity may modulate autoimmune risk, adiponectin and other adipokines were not directly measured in this dataset. The proposed adiponectin-mediated suppression of Th1 pathways remains a speculative mechanism requiring empirical validation.

**Figure 7:**
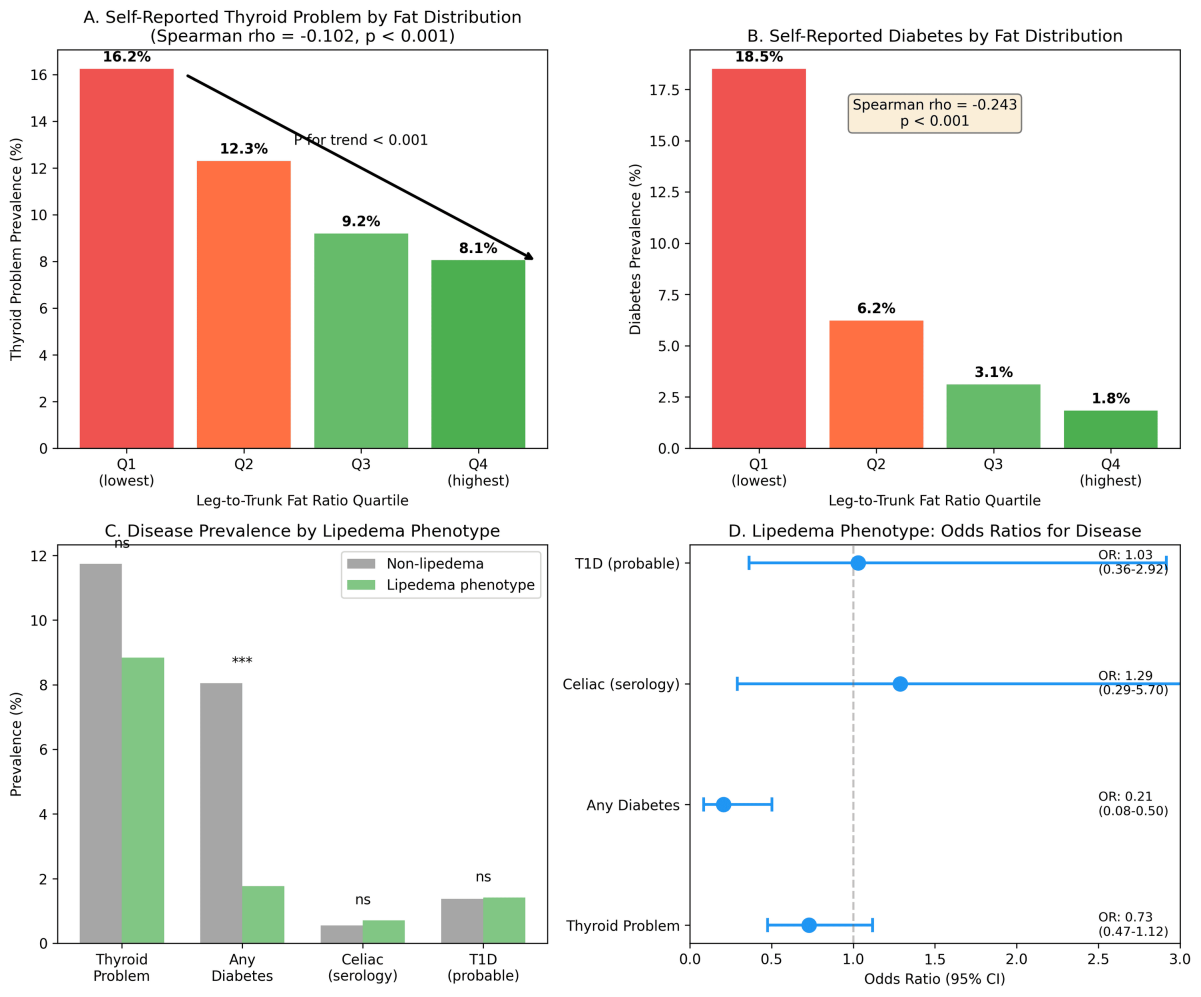
Secondary Analysis: Systemic Immunometabolic Trends and Validation. (A and B) Dose-response relationships: the prevalence of self-reported thyroid problems (A) and diabetes (B) decreases monotonically across quartiles of leg-to-trunk fat ratio (P for trend<0.001 for both). The negative Spearman correlations confirm a graded protective effect of gluteofemoral adiposity. (C) Prevalence comparison: bar chart comparing disease prevalence between women with the lipedema phenotype (green, >90th percentile leg-to-trunk ratio) and controls (gray). The lipedema phenotype group exhibits a drastic reduction in diabetes prevalence. (D) Odds ratios (OR): forest plot showing the odds of having each condition for women with the lipedema phenotype compared to controls. The phenotype is strongly protective against diabetes (OR: 0.21; 95% CI: 0.08-0.50) and shows a protective trend for thyroid disease (OR: 0.73), supporting the hypothesis of a generalized "immunological buffering" effect. ***P<0.001. T1D: type 1 diabetes

Our exploratory analysis showed lower NLR and HOMA-IR in the lipedema phenotype, aligning with the concept of "metabolically healthy obesity" (Table 4 and Figure 4). Although adiponectin was not directly measured, the 44.2% reduction in HOMA-IR serves as a robust physiological proxy, consistent with literature correlating high gluteofemoral adiposity with elevated adiponectin [27-30]. Secondary analysis demonstrated that the lipedema phenotype is associated with lower odds of diabetes (OR: 0.21, p<0.001) and shows an inverse association with thyroid disease (Figure 7), confirming a broadly protective immunometabolic profile.

The "IgG paradox" in lipedema further supports this hypothesis. Previous work demonstrated that while patients with lipedema exhibit widespread food sensitivities, they mount a blunted antibody response, suggesting the systemic downregulation of humoral immunity [31]. Mechanistically, adiponectin reduces CD4+ T cell differentiation into Th1 cells, decreasing interferon gamma (IFN-γ) production [32-38].

The evolutionary shield hypothesis

We have previously proposed that the lipedema phenotype may represent an ancestral, adaptive mechanism for energy conservation and immunological stability [14,39-43]. The present findings, particularly the favorable immunometabolic profile observed in women with disproportionate gluteofemoral adiposity, are consistent with this evolutionary framework, although direct evidence of evolutionary selection remains beyond the scope of epidemiological analysis. In an evolutionary context, storing energy safely in the lower extremities without metabolic penalties would offer survival advantages during reproductive years. This "thrifty phenotype" may be coupled with a downregulated immune surveillance system, naturally suppressing hyper-vigilant autoimmune responses.

Previous studies reported a high prevalence of human leukocyte antigen (HLA)-DQ2 and HLA-DQ8 alleles in patients with lipedema [13]. Our observation of reduced disease prevalence in this same phenotypic group suggests genotype-phenotype decoupling. The lipedema adipose tissue environment may exert a dominant immunomodulatory influence that prevents the clinical manifestation of celiac disease despite genetic susceptibility [13,44].

Clinical implications

The potential immunomodulatory role of gluteofemoral adipose tissue raises important questions regarding large-volume liposuction in lipedema [45]. If this depot functions as an active endocrine organ, as suggested by its association with lower HOMA-IR and NLR, the removal of this depot could theoretically alter the immunometabolic profile described here [6,8,16,17,26,46-49]. While the present cross-sectional data cannot establish causality, these observations provide a compelling rationale for prospective studies incorporating pre- and postoperative immunological monitoring in lipedema surgery registries to evaluate whether fat redistribution alters autoimmune risk profiles, a clinically urgent question given the increasing volume of large-volume liposuction procedures performed worldwide.

Limitations

The primary limitation is the small number of celiac cases (n=11), inherent to investigating a low-prevalence condition (0.5%-1%). While this sample size is expected for hypothesis-generating research, given celiac disease prevalence (~0.5%), all estimates derived from 11 cases are statistically unstable. Small changes in case classification could materially alter the results, and the study was underpowered to detect meaningful differences in phenotype prevalence or dose-response relationships. These findings neither support nor refute the hypothesis; they simply reflect insufficient statistical power to address this question, which remains open for future investigation. The observed difference in gynoid fat (7.4%, p=0.0007), while statistically significant, should be interpreted cautiously given these constraints, even though it intensified in overweight/obese women (8.7%, p=0.005). Stratification by BMI revealed a consistent pattern: 3.4% reduction in normal-weight women, 4.7% in overweight, and 11.3% in obese, suggesting that the celiac inflammatory milieu specifically antagonizes gluteofemoral fat expansion even in energy surplus. The cross-sectional nature precludes causal inference, and the DXA-derived proxy cannot replace clinical lipedema diagnosis. The >90th percentile leg-to-trunk fat ratio threshold may capture normal constitutional variation in fat distribution, such as gynoid obesity or pear-shaped body habitus, rather than true lipedema pathology. This proxy has not been validated against clinically confirmed lipedema cohorts in this dataset, and its sensitivity and specificity for lipedema remain unknown. Validation against clinically diagnosed cases is a necessary step before stronger interpretive claims can be made. Specific inflammatory biomarkers (high-sensitivity (hs) CRP and adiponectin) were unavailable in the 2011-2014 NHANES cycles. Additionally, NHANES does not include the direct measurement of adipokines such as adiponectin or leptin, requiring the use of established proxies (HOMA-IR and body composition measures). Longitudinal studies should track autoimmune health in women undergoing lipedema surgery, and mechanistic studies should profile the adipokine secretome to directly test its suppressive effect on Th1 pathways.

The ethnic composition differed substantially between groups (81.8% non-Hispanic White in celiac versus 36.2% in non-celiac, p=0.003), and the small number of celiac cases precluded robust multivariable adjustment or stratified analyses controlling for ethnicity. The observed associations may therefore be influenced by unmeasured confounders, and this ethnic imbalance warrants particular attention in future studies with adequate statistical power for adjusted analyses.

## Conclusions

This exploratory population-based analysis identified a consistent phenotypic divergence between celiac disease autoimmunity and the DXA-defined lipedema phenotype. Women with celiac disease exhibited significantly lower gynoid adiposity (7.4% reduction, p=0.0007), an effect that persisted and intensified in overweight/obese strata (8.7%, p=0.005), arguing against confounding by leanness. The lipedema phenotype, in turn, was characterized by a distinctly favorable immunometabolic profile, with 44.2% lower HOMA-IR and 7.6% lower NLR. The prevalence of the lipedema phenotype did not differ significantly between groups (p=0.570), and no dose-response was observed (p=0.893); however, with only 11 celiac cases, the study was substantially underpowered for these comparisons. The tissue-level findings, significant, consistent across BMI strata, and biologically coherent, provide stronger evidence than the null prevalence comparison, which would require substantially larger sample sizes to be informative.

These findings provide preliminary support for the "Immunological Shield Hypothesis" at the tissue level and identify gluteofemoral adiposity as a potentially immunomodulatory depot that demands dedicated investigation. The consistency of the gynoid fat finding across BMI strata, combined with the favorable immunometabolic profile of the lipedema phenotype, establishes a foundation for a new line of inquiry at the intersection of adipose biology and autoimmunity. Future studies with clinically confirmed lipedema diagnoses, direct adipokine measurement, and prospective designs, including immunological monitoring in lipedema surgery cohorts, are needed to determine whether this phenotypic divergence reflects a true protective mechanism, a question with significant implications for understanding autoimmune susceptibility and for the clinical management of lipedema. The power calculations presented here (225-600 celiac cases) provide a concrete benchmark for designing such validation studies.

## Figures and Tables

**Figure 8:**
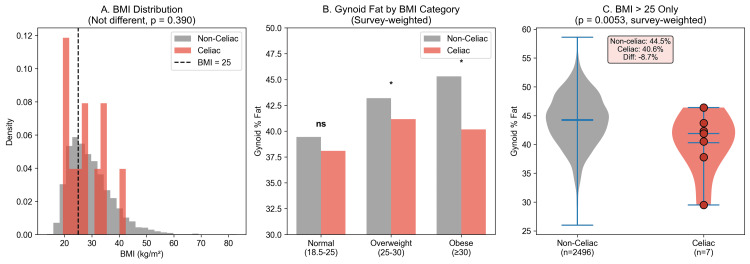
Addressing the Leanness Bias: Gynoid Fat Differences Persist in Overweight/Obese Women. (A) BMI distributions for women with (red) and without (gray) celiac disease are not significantly different (p=0.526), indicating that celiac patients in this population are not systematically underweight. (B) Gynoid percent fat stratified by BMI category. The direction of the difference (celiac lower than non-celiac) persists across all BMI categories, reaching statistical significance in obese women (p=0.039). (C) Comparison restricted to overweight and obese women only (BMI greater than 25 kg/m²). Even among these clearly non-malnourished women, celiac patients have significantly lower gynoid fat (40.6% versus 44.5%, p=0.005). Red dots in panel C represent individual celiac cases. These findings demonstrate that the association between celiac disease and lower gynoid fat is not explained by overall leanness or malnutrition. *Statistically significant. BMI, body mass index; ns, not significant

## Appendices

Sensitivity analysis: Addressing the leanness bias

To ensure that the observed reduction in gynoid fat among women with celiac disease was not an artifact of lower body weight or malabsorption, we stratified the analysis by body mass index (BMI) categories using survey-weighted estimates (Figure 8, Table 5, and Table 6).

**Table 5:** Body Composition in Overweight and Obese Women Only (BMI>25 kg/m²). Data are presented as survey-weighted mean±SD. Analysis restricted to women with BMI>25 kg/m² to rigorously exclude underweight individuals. P-values derived from survey-weighted t-tests. *P-values were derived from survey-weighted t-tests. BMI, body mass index; SD, standard deviation

Variable	Non-celiac (n=2,496)	Celiac Disease (n=7)	Difference	P-value*
Gynoid region percent fat	44.5±4.6	40.6±3.6	-8.70%	0.005
Android region percent fat	43.1±5.5	39.8±4.9	-7.70%	0.109
Leg-to-trunk ratio	0.79±0.21	0.73±0.27	-7.80%	0.435

**Table 6:** Gynoid Percent Fat Stratified by BMI Category (Survey-Weighted). The reduction in gynoid fat associated with celiac disease persists and intensifies in higher BMI categories, demonstrating that the finding is independent of overall leanness. *Statistically significant difference (p<0.05). BMI: body mass index

BMI Category	Celiac (n)	Celiac Gynoid, %	Non-celiac (n)	Non-celiac Gynoid, %	Difference	P-value
Normal (18.5-25 kg/m²)	4	38.1±3.1	1,180	39.4±4.5	-3.4%	0.229
Overweight (25-30 kg/m²)	3	41.2±1.2	985	43.2±4.4	-4.7%	0.024*
Obese (≥30 kg/m²)	4	40.2±4.6	1,534	45.3±4.4	-11.3%	0.039*

Secondary analysis: Validation of the Immunometabolic Shield Hypothesis

To further validate the biological plausibility of the "lipedema phenotype" (defined as leg-to-trunk fat ratio of >90th percentile) as a protective immunometabolic state, we assessed its association with other systemic conditions (Table 7, Figure 5, and Figure 7).

**Table 7:** Association of Lipedema Phenotype With Metabolic and Autoimmune Conditions. OR: odds ratio

Condition	Non-lipedema (n=2,547)	Lipedema Phenotype (n=283)	OR (95% CI)	P-value
Diabetes (any type)	8.10%	1.80%	0.21 (0.08-0.51)	<0.001
Self-reported thyroid problem	11.70%	8.80%	0.73 (0.47-1.13)	0.168
